# The Influence of Collaboration and Culture on the IKEA Effect: Does Cocreation Alter Perceptions of Value in British and Indian Children?

**DOI:** 10.1037/dev0001321

**Published:** 2022-04

**Authors:** Lauren E. Marsh, Joanna Gil, Patricia Kanngiesser

**Affiliations:** 1 School of Psychology, University of Nottingham; 2 Faculty of Education and Psychology, Freie Universität Berlin; 3 School of Psychology, University of Plymouth

**Keywords:** IKEA effect, psychological ownership, collaboration, cross-cultural

## Abstract

Creating objects can increase our evaluation of them, even when we compare them to physically identical copies (IKEA effect). Here we evaluate the influence of collaboration on the IKEA effect in two societies—the United Kingdom and India. One hundred twenty-eight 5-to-6-year-old children (48% female, 50% British middle class, 50% Indian middle class) assembled toys in pairs. Half of the children collaborated to assemble a single toy and half assembled their own toy. In both societies, children demonstrated an IKEA effect (η^2^_p_ = .19), valuing their own creation over an identical copy. This was the case regardless of whether children collaborated or worked independently. In summary, it seems that the IKEA effect is a potent bias that is present in diverse societies and is insensitive to others’ contributions in a collaborative environment.

Our valuation of an object is rarely based just on its utilitarian functions. Instead, we are heavily biased by the history we can attach to an object: who made it, who owned it previously, or how we acquired it (Newman & Bloom, 2012; Newman et al., 2011). The act of creating an object leads the creator to attribute a higher value to that item, compared with a scenario in which they had acquired it by other means. This so called “IKEA effect” (Norton et al., 2012) has been demonstrated in adults from Western and Northern Europe and North America in numerous contexts including customization of design products (Franke et al., 2010), and food production (Dohle et al., 2014; Radtke et al., 2019; Troye & Supphellen, 2012), but also in the construction of utilitarian, noncustomizable items (Norton et al., 2012; Mochon et al., 2012).

## Theoretical Accounts of the IKEA Effect

Several researchers have proposed psychological drivers of the IKEA effect. Perhaps the effort we invest in creating something leads us to overvalue reward for our efforts (effort justification account; Norton et al., 2012). Alternatively, the created object may function as a trophy, signaling pride and competence to others (Bühren, & Pleßner, 2014, 2020; Mochon et al., 2012). Recently, developmental work has indicated that neither of these accounts adequately explain the IKEA effect, as evaluations of creations did not differ as a function of the amount of effort invested, or whether the object was prominently displayed to others (Marsh et al., 2018).

The present study is embedded within a third account that proposes that psychological ownership leads to increased valuation (Walasek et al., 2017). North American adults are more likely to attribute ownership to people who have contributed labor to the creation of an object, compared with people who have invested ideas (Burgmer et al., 2019). Object creation also led British adults to report increased subjective feelings of ownership (Walasek et al., 2017). Similarly, developmental research has shown that British children will transfer ownership rights to the creator of an object (Kanngiesser & Hood, 2014; Kanngiesser et al., 2010) and North American children show greater respect for ownership of objects that have been made, compared with objects that have been found (Davoodi et al., 2020). Thus, it seems that throughout development, creating an object results in strong ownership ties between the creator and the object, in the eyes of both the creator and third parties.

The upshot of increased feelings of ownership is increased valuation. The Endowment Effect is a bias in which adults value items in their possession over identical, unowned items (Kahneman et al., 1990; Thaler, 1980). This bias is also present early in development and by age 5, British and American children are biased by IKEA effects (DeJesus et al., 2019; Marsh et al., 2018) and Endowment effects (Harbaugh et al., 2002; Hood et al., 2016) as both self-created and self-owned items are valued over identical copies. While the underlying mechanisms are still debated, it has been proposed that the Endowment Effect is present in societies that are market integrated (Apicella et al., 2014), and is driven by feelings of ownership for items in our possession (Morewedge & Giblin, 2015). Indeed, a study by Sarstedt et al. (2017) provides preliminary evidence in support of this claim by demonstrating for Northern European adults that the value a creator placed on their creation was partially mediated by their self-reported feelings of psychological ownership. However, these effects were based on a correlational design, capitalizing on individual differences in the valuation of creations. Further evidence indicates that Endowment Effects can be strengthened through self-construal priming in North American adults (Maddux et al., 2010) and British children (Weltzien et al., 2018).

## Collaboration, Collective Ownership, and Object Valuation

Psychological ownership appears to be a direct consequence of object creation, making it challenging to tease apart labor and ownership accounts of the IKEA effect experimentally. However, one possible avenue is to investigate the impact of collaboration on the IKEA effect as cocreation should lead to collective ownership claims (Pierce & Jussila, 2010; Verkuyten & Martinovic, 2017). To date, no empirical work has directly tested the extent to which collaborative versus individual creation has an impact on the value attributed to objects. Additionally, there is, to our knowledge, no research into how cultural context affects the IKEA effect and whether it is subject to cultural variability. We speculate that collaborative efforts may result in a weaker IKEA effect where ownership is shared among multiple creators, especially in societies in which autonomy and independence are highly valued (Kagitcibasi, 2005; Markus & Kitayama, 1991). The present study tests this hypothesis in a developmental, cross-cultural sample.

Collaborative compared with individual activities have an impact on a range of behaviors early in childhood. For example, German preschoolers who engage in cooperative activities compared with individual activities more often help their partner achieve their goals (Hamann et al., 2012), they make more attempts to reengage partners that have left a joint activity (Gräfenhain et al., 2009), and are more likely to resist temptations to abandon a collaboration (Kachel & Tomasello, 2019). Cooperative versus individual activities also determine how preschoolers divide resources. American preschoolers show merit-based sharing after individual work (Kanngiesser & Warneken, 2012), but when rewards were obtained through collaboration German preschoolers shared them equally (Hamann et al., 2011; Warneken et al., 2011). Sharing also increases after working toward a joint goal (Canadian preschoolers; Corbit, 2019) and older children (in rural Canada and rural India) will reject advantageous inequity after collaboration but not after parallel work (Corbit et al., 2017). Thus, young children from Europe and North America (and rural India) are sensitive to experimental manipulations of collaboration, and such activities can promote prosocial tendencies, such as sharing. Little is known, however, about the impact of collaboration on perceived ownership in creative activities.

## Societal Variation in Endowment and IKEA Effects

Much of what we know about the IKEA effect comes from studies conducted in North America and Western and Northern Europe. Therefore, it is difficult to ascertain whether the bias perpetuates broadly across human societies or if it is limited to some societies. This has been identified as a key issue in the field of developmental psychology (Nielsen et al., 2017). There is some cross-cultural evidence that the Endowment Effect varies between Canadian, Chinese and Japanese university students (Maddux et al., 2010). Yet, to elicit Endowment effects participants are usually allocated objects and there is no effort involved in obtaining or creating them (IKEA effect), but see Bühren and Pleßner (2020) for conditions where effort and labor are directly compared. Cross-cultural, developmental work on children’s ownership understanding suggests that the investment of effort and labor acts as a strong signal of ownership that is present across a range of diverse societies and socioeconomic status (SES) groups from the preschool years (Kanngiesser et al., 2014; Rochat et al., 2014). This suggests that the IKEA effect may be less impacted by cultural context than Endowment effects.

## The Present Study

Here we examine IKEA effects for individually and collaboratively created objects in children from two distinct societies. Based on the reviewed literature, we hypothesize that children from both the United Kingdom and India will show a robust IKEA effect for individually created objects (Kanngiesser et al., 2014; Marsh et al., 2018; Rochat et al., 2014). However, we speculate that cultural differences may emerge when children are asked to cocreate an object. We hypothesized that children from the United Kingdom would demonstrate a weaker IKEA effect for a cocreated object, compared with an individually created object because children in Western societies tend to be socialized to value autonomy and independence over relatedness (Kagitcibasi, 2005; Keller et al., 2010). In contrast, we hypothesized that this dilution of the IKEA effect due to collaboration would not be present in the Indian children due to a greater societal emphasis on relatedness than in urban Western samples (Keller et al., 2010; Kärtner et al., 2016). In Phase 1 of this study, one hundred twenty-eight 5-to-6-year old children from the United Kingdom and India individually completed a shopping task that elicited relative worth judgements for a series of small toys, including a monster finger puppet (as in Marsh et al., 2018). In Phase 2, children were then paired with another child from their class and were randomly assigned to a collaborative or an individual build condition. In the collaborative build condition, children worked together to make a monster finger puppet, identical to the one used in the shopping task. In the individual build condition, children sat next to each other, but made separate monsters. Following the build task, children completed the shopping task again to value the monster they made as well as the identical monster.

## Method

### Participants

One hundred twenty-eight 5-to-6-year old children were included in this study. Sixty-four British children (*M*_age_ = 75 months, range = 67–85 months, 32 female) were recruited from three primary schools in Bristol. Sixty-four Indian children (*M*_age_ = 76 months, range = 69–82 months, 30 female) were recruited from two English-medium primary schools in Pune. Bristol is a medium-sized urban city in the South-West of England, with an estimated population of 460,000 people. Main employing industries include Retail and Health and Social Care. The city is reasonably diverse with 22% non-White British residents. Pune is a large-sized urban city in the West of India, with an estimated population of 4 million residents. Main industries include IT and manufacturing. The sample size was specified in the study preregistration on the Open Science Framework (OSF, https://doi.org/10.17605/OSF.IO/T58Z9) and was based on previous studies (Marsh et al., 2018).

An additional 25 Indian children completed Phase 1, but not Phase 2 because they did not pass valuation training (*n* = 11), were absent on the second testing day (*n* = 8), or it was not possible to match them to a same-sex classmate for Phase 2 (*n* = 6). An additional 13 British children completed Phase 1 but not Phase 2 because they failed to pass the valuation training (*n* = 4), they were absent on the second testing day (*n* = 5), or it was not possible to match them to a same-sex classmate for Phase 2 (*n* = 4). All parents gave written, informed consent before participation. Testing was completed in English in a quiet classroom within the child’s school. Indian children were proficient in speaking English. This study, entitled “Is the IKEA effect prevalent across cultures,” received ethical approval from the ethical review board at the University of Bristol (ID: 31031632882).

### Procedure

**Figure 1 fig1:**
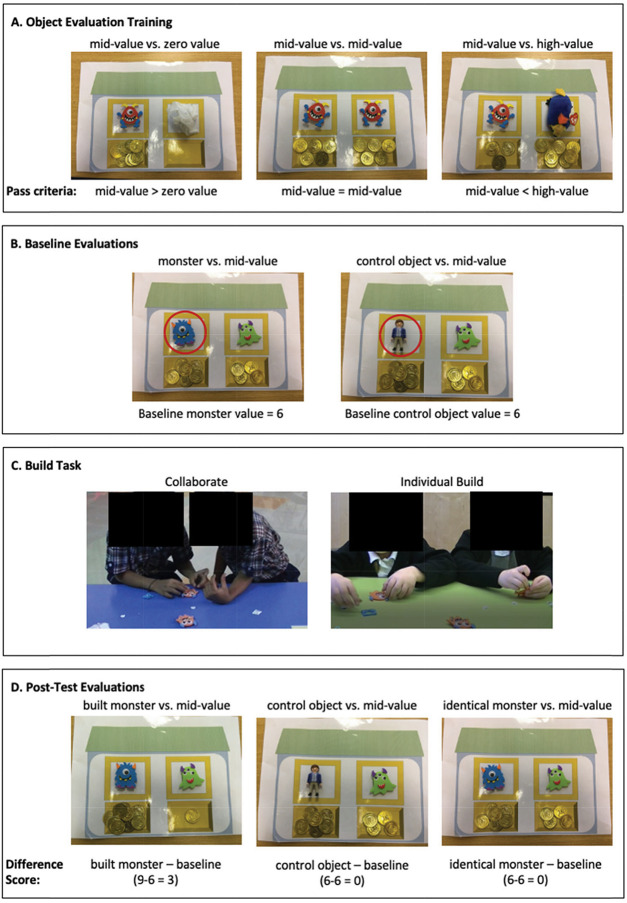
Overview of the Study Procedure and Materials *Note*. Panel A depicts the training trials and pass criteria that children needed to attain to be included in the study. Panel B shows how we elicited baseline valuations. The red circle indicates the item of interest. Panel C shows different interactions elicited by the collaborative and individual build conditions. Panel D shows how post-test evaluations were elicited and gives an example difference score calculation. See the online article for the color version of this figure.

This study was preregistered on the OSF (https://doi.org/10.17605/OSF.IO/T58Z9). The study comprised of two phases, completed on consecutive days. In Phase 1, we tested children individually. First children completed a friendship nomination task where we asked them to name all their friends in their class. Children could nominate as many friends as they liked. The purpose of this was to assess the social network position of children within each class. Next, we trained children in an object evaluation task, as reported in Marsh et al. (2018). After an initial demonstration by the experimenter, children were presented with pairs of items and instructed to assign 10 coins between them, to indicate their relative worth (“How much will you pay for each item?”). Training pairs included two identical midvalue items, a high-value item with a midvalue item, and a midvalue item with a zero-value item (see Figure 1). To pass training, children had to assign more coins to the more valuable item, and an identical number of coins to the identical items. Finally, we used the same procedure to elicit baseline object evaluations for a foam monster (identical to the one built in Phase 2) paired with a midvalue item, and a control object (a plastic figurine) paired with the same midvalue item. Children were instructed to assign all 10 coins for each object pair. A maximum value of 10 coins, and a minimum value of 0 coins could be assigned to any item.[Fig fig1]

In Phase 2, we tested children in pairs. Dyads were always same-sex, from the same class, and as closely matched on baseline valuations of the foam monsters as possible (*M*_diff_ = 1.14 coins). We did not select dyads on the basis of friendship nominations, although we assessed after testing whether children were paired with a friend or not. Twenty-three (36%) Indian children and 22 (34%) British children participated with someone they nominated as a friend. We randomly assigned dyads to one of two between-subjects conditions: collaborative build or individual build. In both build conditions, children sat next to each other and received pieces to make a foam monster (identical to the monster valued at baseline in Phase 1). In the collaborative build condition, we gave each child half of the pieces and instructed them to make one monster together. In the individual build condition, we gave children pieces to make a monster each. The experimenter turned away and pretended to be busy while the children built the monsters. Once finished, each child individually completed posttest evaluations with the experimenter while the other child completed a coloring task with headphones on. We elicited posttest evaluations for the monster they had built, the control object, and the identical monster valued at baseline, using the object evaluation task from Phase 1. In each case, we paired the target item with the same midvalue item used in Phase 1. The experimenter verbally labeled the monsters as “this is the one you built” and “this is not the one you built” so that children could keep track of their creation. We counterbalanced the order of the own-built and identical monster valuations, with the control object always valued second so that children never valued the two monsters sequentially. Finally, to assess monster preference, the built monster and the identical monster were placed side-by-side and we asked children which of the two monsters they liked best and why. Again, the experimenter verbally labeled the monsters for the child to avoid mixing them up. After both children had completed the posttest valuations, they attached their monster to a display on the wall of the classroom. We videotaped both study phases so that it was possible to check the valuation responses and to complete behavioral coding of the interactions between children during the build task.

### Data Coding and Analysis

We coded all responses live and from videotape. There were no discrepancies between live coding and video coding for the valuation responses. Analysis of covariance (ANCOVA) analyses were conducted using SPSS and GLMM analyses were conducted using the lme4 package in R.

#### Valuation of the Built Object

Difference scores for the valuation of the built monster and the identical monster were calculated by subtracting the baseline monster value from the value assigned to each monster at posttest. Each item could be valued between 0 and 10, so difference scores could range from 10 to −10, with higher values indicating an increase in valuation over the experiment. To analyze difference scores, we used an ANCOVA, with object (built, identical) as a within-subjects factor, and condition (collaborative, individual), society (United Kingdom, India) and gender as between-subjects factors. We entered age in months as a covariate.

#### Valuation of the Control Object

A difference score was calculated for the control object by subtracting the value of the control object at baseline from the value of the control object at posttest. We used a separate ANCOVA to analyze difference scores for the control object. This was to ensure that children’s valuations did not generally change over the course of the experiment as a result of one of the experimental manipulations. Condition (collaborative, individual), society (United Kingdom, India), and gender were entered as between-subjects factors and age in months was entered as a covariate.

We preregistered these analyses on the OSF (https://doi.org/10.17605/OSF.IO/T58Z9). However, our preregistration did not account for the nested structure of the data (i.e., children providing data within a dyad are not independent from one another). As such, we ran additional GLMMs that take this into account. These analyses provided comparable results and are reported in online supplemental materials.

#### Friendship Nominations

As rates of parental consent per class were low in the United Kingdom (<30% per class) it was not possible to use friendship ratings to assess the social network position of the children taking part, as originally planned. Instead, we used friendship nominations to determine whether children were paired with someone they consider to be their friend or not. To assess whether being paired with a friend influenced the IKEA effect in each condition and across societies, we conducted an additional ANCOVA.

Children either picked their own creation (*n* = 81), the identical monster (*n* = 32), or stated that they were the same (*n* = 15). Monster preference was analyzed using a GLMM to assess whether the number of children selecting their own creation varied by society or collaboration condition. A full model including society, collaboration condition, the interaction between society and collaboration condition, participant gender and age was compared with a null model including only gender and age using likelihood ratio tests.

### Behavioral Coding

Behavioral coding of the build interaction was completed as a manipulation check, to assess whether children engaged in different interaction styles in collaborative and individual build conditions (Little et al., 2016). The length of time that children engaged in individual activity (partners engaged in individual tasks) vs triadic activity (partners engaged in joint tasks, by looking at or touching the same pieces of a monster) was coded from video. A second coder scored 21% of the videos for reliability purposes. Agreement between coders was 97% for individual activity (κ = .28), and 93% for triadic interactions (κ = .68). The kappa value for individual activity is low despite very high rates of agreement, because our coding was heavily skewed as both coders coded some categories particularly frequently and others very rarely (see online supplemental materials for further details). Time spent in triadic interactions was compared across condition (collaborative, individual) and society (United Kingdom, India) using GLMM. The analysis of behavior during the build task was not preregistered and is exploratory.

All data have been made publicly available at the OSF and can be accessed at: https://osf.io/kus8e/. Materials and analysis code for this study are available by emailing the corresponding author.

## Results

### Valuation of the Built Object

**Figure 2 fig2:**
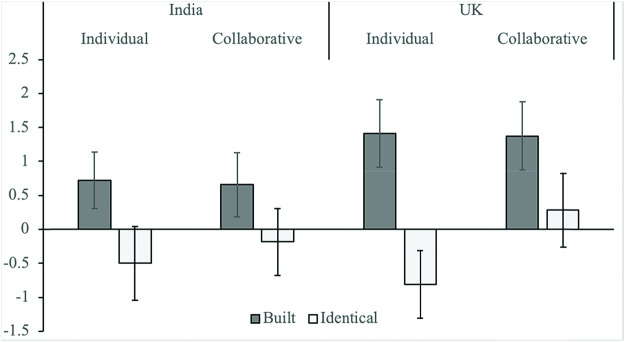
Value Change of the Own-Built Monster (Dark Bars) and the Identical Monster (Light Bars) as a Function of Collaboration Condition and Society *Note*. Error bars represent ±1 *SEM*.

In evidence of an IKEA effect, children increased the value of the monster that they had built (*M* = 1.06, 95% confidence interval, CI [.60, 1.52]) more than an identical monster which they had not built (*M* = −.27, 95% CI [−.77, .23], *F*(1, 119) = 28.23, *p* < .001, η^2^_p_ = .19, Figure 2 and Figures S1 and 2 for raw data). There was no main effect of condition; children overvalued the monsters regardless of whether they had built a monster individually (*M* = .25, 95% CI [−.33, .83]) or collaboratively (*M* = .54, 95% CI [−.04, 1.11], *F*(1, 119) = .47, *p* = .494, η^2^_p_ = .004). There was also no main effect of society on valuations (*M*_United Kingdom_ = .54, 95% CI [−.04, 1.12], *M*_India_ = −.24, 95% CI [−.34, .82], *F*(1, 119) = .54, *p* = .465, η^2^_p_ = .004). Contrary to our hypotheses, the three-way interaction between condition, society, and object (*F*(1, 119) = .67, *p* = .414, η^2^_p_ = .006, BF_01_ = .013), and the two-way interactions between condition and object (*F*(1, 119) = 2.08, *p* = .152, η^2^_p_ = .017), and society and object (*F*(1, 119) = 1.57, *p* = .213, η^2^_p_ = .013) were not significant. This indicates that the IKEA effect was not modulated by collaboration, and was consistent across the two societies in our study. There was a significant effect of gender (*F*(1, 119) = 4.61, *p* = .034, η^2^_p_ = .037), in which females (*M* = .84, 95% CI [.25, 1.43]) increased the value of both monsters more than males (*M* = −.06, 95% CI [−.63, .52]), but gender did not interact significantly with any other variable (all *F*s < 2.42, all *p*s > .123).[Fig fig2]

### Valuation of the Control Object

Children who collaborated decreased the value of the control object (*M* = −1.08, 95% CI [−1.67, −.48]) more than children who completed independent builds (*M* = −.15, 95% CI [−.75, .45], *F*(1, 119) = 4.73, *p* = .03, η^2^_p_ = .038). There was no significant effect of society (*M*_United Kingdom_ = −.71, 95% CI [−1.31, −.11], *M*_India_ = −.52, 95% CI [−1.12, .08], *F*(1, 119) = .21, *p* = .652, η^2^_p_ = .002), and no significant interaction between condition and society (*F*(1, 119) = .298, *p* = .586, η^2^_p_ = .002) on control object valuations.

### Friendship Effects on the Valuation of Built Objects

There was no significant effect of friendship (*F*(1, 111) = .17, *p* = .683, η^2^_p_ = .002), and no significant interaction between friendship and other experimental variables on valuation of the monsters (all *F*s < 2.24, all *p*s > .137). Children valued the monsters in the same way, regardless of whether or not they were paired with a friend during the build phase of the task.

### Monster Preference

In the individual build condition, 24 (75%) British and 18 (56%) Indian children selected the monster they had created as the preferred option. In the collaborate condition, 20 (63%) British and 19 (59%) Indian children preferred the monster they had built. There was no significant difference between the full and the null model (χ^2^(3) = 3.95, *p* = .267), indicating that society, collaboration, and the interaction term had no explanatory effect on monster preference.

### Behavioral Interactions During the Build Task

**Figure 3 fig3:**
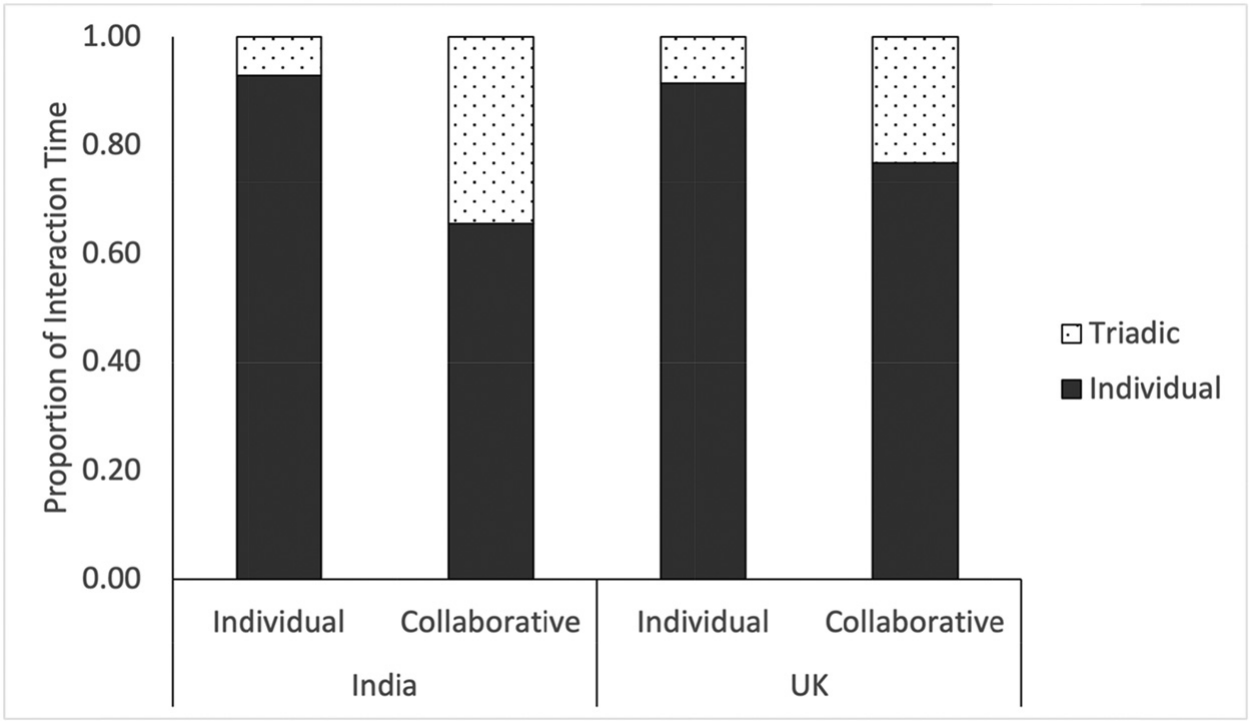
Proportion of Time Spent Engaged in Individual (Dark Bars) and Triadic (Light Bars) Interactions as a Function of Collaboration Condition and Society

The full model had the best fit to the data (see Figure 3). Children from India engaged in proportionally more triadic interactions than children from the United Kingdom (χ^2^(2) = 10.59, *p* = .005). Children also engaged in a higher proportion of triadic interactions in the collaborative condition, compared with those who built the monsters individually (χ^2^(2) = 32.49, *p* < .001). These main effects were qualified by an interaction between condition and society (χ^2^(1) = 5.79, *p* = .016). Pairwise Tukey corrected comparisons revealed that Indian children engaged in a higher proportion of triadic interactions in the collaborate condition, compared with children from the United Kingdom (*t*(25) = 3.08, *p* = .024). There was no difference in the proportion of triadic interactions between Indian and British children in the individual build condition (*t*(25) = .53, *p* = .950).[Fig fig3]

## Discussion

This study aimed to assess the strength of the IKEA effect for objects that children built collaboratively vs individually and to examine this bias in children from two different societies. We found robust IKEA effects in all conditions of this study. Contrary to our predictions, collaborative work resulted in children valuing their creations just as highly as creations built individually. In addition, this bias was similarly present in children from the United Kingdom and children from India and did not depend on whether the child was paired with a friend for the task. Exploratory analyses revealed variation in behavioral performance of collaborative activities across society and condition. We discuss the implications for each of these findings in the sections below.

### Societal Comparisons of Valuation and Collaboration

There was no societal variation in the size of the IKEA effect demonstrating that children in the United Kingdom and India alike value their own creations more than identical items created by someone else. As predicted, this study demonstrates that the IKEA effect is robust and replicable across different societies. However, we did expect the collaborative condition to draw out societal differences in this task. Specifically, we predicted that British children would value their individual creations more than the product of collaboration as individual efforts and achievements are regarded more highly. In India we predicted that collaboration would have less influence on the size of the IKEA effect. The lack of societal variation could be another demonstration of the robustness of the IKEA effect as its manipulation has proved elusive in other developmental studies (Marsh et al., 2018).

Alternatively, this null result could reflect a methodological issue with the collaborative condition in which children do not actually engage in collaborative activity in this task. Instead, children could be completing the build in serial, rather than jointly engaging with their partner. To rule out this possibility, a post hoc behavioral coding analysis of children’s interactions during the task was conducted. As expected, children engaged in more triadic interaction during the collaborative build condition, compared with the individual build condition. This indicates that the collaboration manipulation was effective in altering behavioral interactions between children. In addition, this analysis revealed societal differences in interaction style during the collaborative build task as Indian children engaged in proportionally more triadic interactions than British children. Previous studies have found differences in children’s cooperation styles depending on their parents’ level of schooling (Alcalá et al., 2018; Chavajay & Rogoff, 2002; Correa-Chávez, 2016)—though children in both of our samples came from urban middle class families. This tentatively suggests that factors other than parental education may also play a role in collaboration styles. However, it is important to note that this analysis was exploratory and based on a limited number of codeable videos that have an unbalanced number in each condition. We believe that these findings are interesting but remain cautious in our interpretations.

### The Link Between Collaboration and Value

We hypothesized that collaborative creation of an item would induce collective ownership in the creators, and that this would weaken the IKEA effect. Contrary to predictions, children demonstrated an equally sized IKEA effect, regardless of whether they collaborated or not. There are three possible explanations for this result. First, the collaboration manipulation may have been ineffective at inducing collaboration. Second, collaboration does not induce collective ownership, or third, the product of collaboration is valued equally to an individual creation. Each of these explanations will be discussed below.

With regards to the effectiveness of the collaboration manipulation, the behavioral data contribute to our understanding by showing that our experimental manipulation did have an effect on children’s behavior. As mentioned above, children in the United Kingdom and India engaged in more triadic interactions in the collaborative condition, compared with the individual condition. This indicates that the children in the collaborative condition did treat the task as a shared endeavor, by either helping or watching their partner as they contributed to the build task.

Although not directly tested, we believe that it is equally unlikely that collaboration failed to induce collective ownership in the creators. Previous work has demonstrated that children as young as 3-years-old will share the spoils of collaboration equally, demonstrating collective ownership of their reward (Warneken et al., 2011). Thus, it seems reasonable that the same might be true in this case.

The final explanation that the product of collaboration is valued as highly as an individual creation, is the most plausible explanation in this case. One previous study has investigated the parameters of collective ownership in young children (Huh & Friedman, 2017). They reported that children aged 3-to-6-years understood that property could be owned by groups, and that group ownership conferred privileged access over nongroup members, but also limited rights in comparison with sole ownership. Therefore, a complex understanding of both the benefits and limitations of collective ownership should be possible for the children in the present study. However, it seems that despite this appreciation, collective ownership as a result of collaboration does not limit product valuations. Further confirmatory work is needed to establish this finding and to explore the limits of collaboration and linked value. For example, does collaboration dilute the value of an item with increasing numbers, or does the identity of the collaboration partner affect valuations? With regards to the latter, being paired with a nominated friend had no impact on the value of creations, although other factors such as perceived similarity might have a greater impact. Future work could also assess the extent to which collaboration influences the IKEA effect in adults. These questions are of importance for organizations who might be seeking to promote value linked to ownership and collaboration (i.e., the use of company shares for employees, or the use of community art projects to deter vandalism). Additionally, in our current “disposable society” there is value in exploring ways to promote the value of objects through creative input, as this might provide a mechanism for promoting sustainable product use (i.e., reusable personalized coffee cups) or other sustainable behaviors linked to psychological ownership (Preston & Gelman, 2020).

To conclude, constructing an item leads to a potent preferential bias toward it (the IKEA effect) that is present early in development. This work is the first to demonstrate that this preferential bias can be the result of individual or collaborative activity, and has been identified in two different sociocultural contexts.

## Supplementary Material

10.1037/dev0001321.supp
