# Supplementary material for: The Influence of Collaboration and Culture on the IKEA Effect: Does Cocreation Alter Perceptions of Value in British and Indian Children?
Source: Dev Psychol. 2022 Apr;58(4):662–70. doi: 10.1037/dev0001321 (PMC8958763; doi:10.1037/dev0001321)
Supplement: Supplementary file 1 [file DEV-2021-4186_Supplemental_Materials.docx]

Supplementary Information for

The influence of collaboration and culture on the IKEA effect: does co-creation alter perceptions of value in British and Indian children?

**Testing Script**

*Training phase (individual testing, day 1):*

“We are going to play a shopping game today. Can you see my shop here? There are two windows in my shop. I am going to put some different things in the shop windows and then we can use these coins to show each other how much we think the things are worth.”

“First of all, can you count out the coins and tell me how many there are?” [E gives the coins to the child to count, correct the child if they count wrong].

“That’s right, there are 10 coins. Now I’ll put some things in the shop windows and I’ll start by showing you how much I think they are worth. When I have had a few turns it will be your turn and you can show me.” [E brings out 2 identical toys]

“First of all I have these two things for the shop. Can you see they are exactly the same so I think they should be worth the same amount of coins? I think they are worth 5 counters each” [E places the two identical monsters in the shop windows and 5 counters in front of one toy and 5 counters in front of the other toy]. “Can you see – because they are just the same, I’ve given them the same amount of coins”.

“Now if I change the things in the shop window, I have these two things – a monster and a monkey. I like this one the best, it’s really nice. Because I like this one most, it should be worth more coins. I think it is worth 7 coins. I think this one is only worth 3.” [E distributes coins between the two non-identical toys]. “Do you see – because I like this one best, I’ve given it more coins”.

“Last of all, I have these two things for the shop window – a monster and a piece of card. Hmmm…this is just a piece of rubbish – it’s not really worth anything. So this time, I think the monster is worth all 10 coins, and the card isn’t worth any.” [E places all 10 counters in front of the desirable toy and none in front of the rubbish].

“Now, do you think you could tell me how much you think some different things are worth?”

[E puts pairs of items in the shop windows and waits for the child to distribute the coins between each pair]

“How many coins do you think these things are worth?”

These item pairs were:

Training pairs (order counterbalanced):

- two identical red monsters (not used in the rest of the experiment)
- one red monster and a small cuddly toy (obviously nicer and more valuable than the monster)
- one red monster and a piece of rubbish

*Baseline ratings:*

“How much do you think these two things are worth?”

Baseline ratings (order counterbalanced):

- a blue monster (to be used in the build task) and a green monster (labelled as the mid-value item in the manuscript for clarity)
- the control object (small plastic figure) and a green monster

*Building Phase (paired testing, day 2):*

Collaboration instructions: “I have the pieces so that together you can make a monster just like this one [E holds up the blue monster from the baseline rating task]. Here is the body. You can have these pieces and you can have these pieces. When you are finished you can stick the monster on the wall with the other ones here.”

Individual build instructions: “I have the pieces so that you can each make a monster just like this one. You can have these pieces and you can have these pieces. When you are finished you can stick the monster on the wall with the other ones here.”

[E holds up the build monster, starts the timer and gives the child the body of the monster. In the collaboration condition, children will be given half the pieces each in the own build condition, children will be given all the pieces to make their own monster. E then turns her back and pretends to be busy until the build task is complete].

“Great is that finished now?” [E stops the timer and tidies away spare materials]

[E asks one child to wait on the other side of the table with some headphones on whilst the other child does the shop game behind a screen.]

*Post-interaction ratings:*

“Now, do you remember the shopping game we played yesterday? Can you tell me how many coins you think these things are worth?”

[E places pairs of objects into the shop and hands the child the coins.]

“The [first/last] pair of items for the shop are the blue monster that you [built / did not build] and the green monster. How much do you think these things are worth?”

“Next in the shop we have this plastic man and the green monster. How much do you think these things are worth?”

Post-Interaction pairs (order of built and identical monsters counterbalanced)

- Blue built monster and green monster (mid-value item)
- Control object and green monster
- Blue identical monster and green monster

*Monster Preference Task:*

E places own built and identical monsters in the shop windows and asks two questions

“This is the monster that you built, and this is the monster that you did not build. Which one do you like best?”

“Why?”

**Details of Behavioral Coding**

We first identified videos that could be used for behavioural coding. We excluded videos, in which the majority of the time one or more of the following criteria applied: (a) children’s eyes were cut-off and gaze direction could not be judged, (b) the experimenter was constantly interacting with children (speaking, handing pieces), and (c) one or both children were often absent in the frame or distracted. In total, there remained 34 codeable videos (Pune: 15 videos, 11 in the collaborative condition and four in the individual condition; UK: 19 videos, eight in the collaborative condition and 11 in the individual condition).

We then coded the overall length of the building interaction, starting when all pieces of the monster were on the table and the experimenter left the frame and ending when the task was completed. The main behaviour of interest was whether children engaged in individual or triadic interactions (Little et al., 2016). We used the following coding scheme to score the duration of children’s engagement (only durations >1sec were scored; see Table S1):

- Individual engagement: Engagement was coded as (a) *physical individual engagement* when one child was in physical contact with the monster or parts of it or as (b) *visual individual engagement* when one child looked at the monster or parts of it. There was no engagement from the other child.
- Triadic engagement: Engagement was coded as (a) *physical-physical triadic engagement* when both children were in physical contact with the *same* monster or the *same* parts of the monster, as (b) *physical-visual triadic engagement* when one child was in physical contact with the monster or parts of the monster and the other child observed looked at the same monster or monster parts, and as (c) *visual-visual triadic engagement* when both children looked at the same monster or parts of the monster, there was no physical contact between the monster and either of the children.

Behaviours could occur in parallel. For example, both children could be simultaneously engaged in individual-physical with their own monster, or one child could be holding on to her own monster while attending to what her partner did with the partner’s monster (this would be scored as physical individual and visual-physical triadic). We coded video segments as take-out’s (e.g. not to be included in analyses) when the experimenter briefly interacted with the children or when children’s eyes, heads or hands were not visible or they were distracted. A second coder scored seven videos (21%) for reliability purposes (three videos from India, 4 videos from the UK; 4 collaborative, 3 individual). Agreement between coders was 97% for individual interactions (kappa = .28), and 93% for triadic interactions (kappa = .68). The kappa value for individual interactions is low despite very high rates of agreement, because our coding was heavily skewed as both coders coded physical individual engagement very frequently (175/180 and 178/180 codes, respectively) and visual individual engagement almost never (1/180 codes).

The proportion of time that children engaged in individual vs. triadic interactions was compared across condition and society. These data were analysed with GLMMs, using the lme4 package in R. We constructed a full model by assessing whether condition, society, gender, and the interaction between condition and society predicted the proportion of triadic engagement. Within this model, we included a random intercept for each dyad. We compared this to a null model, containing only gender and the random intercept, and a reduced model in which we only removed the interaction term.

Table S1: Details of behavioural coding scheme applied to video data.

| Maincode | Subcode | Subsubcode | Description |
| --- | --- | --- | --- |
| phase  (duration) | start |  | Start of relevant phase: all pieces of the monster are on the table and experimenter finished interacting with the children. |
|  | stop |  | End of relevant phase: when one child leaves the frame and task (gluing all the pieces to the monsters body) is completed; when experimenter asks if the children are finished and they agree; when it is verbally expressed that the task is finished. |
| take-out’s  (duration) | experimenter |  | Whenever experimenter interacts with the children, this is coded as experimenter interaction, none of the other codes apply; whenever the experimenter speaks to the children (not the children to the experimenter); whenever the experimenter places hands in front of the child as they help; Start: when the experimenter first moves/enters the frame; Stop: when the experimenter finishes moving out of view again. Only code if the duration of the event is >1 sec. |
|  | not-codable |  | Children and their eyes/heads and hands are not clearly visible; they are clearly distracted by things other than the experiment; if this happens too often, the whole video is considered non-codable. Only code if the duration of the event is >1 sec. |
| engagement  (duration) | individual | physical | One child is in physical contact with the monster or parts of it (child doesn’t have to look at the monster/monster-parts); *no* engagement from the other child. Only code if the duration of the event is >1 sec. |
|  |  | visual | One child looks at the monster or parts of it, *no* engagement from the other child. If eyes are a little bit obscured, but the segment is still codable, go off of ‘head turn’. Only code if the duration of the event is >1 sec. |
|  | triadic | physical-physical | *Both* children are in physical contact with the *same* monster or the *same* parts of the monster (children don’t have to look at the monster/monster-parts). Only code if the duration of the event is >1 sec. |
|  |  | physical-visual | *Only one* child is in physical contact with the monster or parts of the monster and the other child observes/looks (but without physical contact to the monster or parts of the monster that the other child is interacting with). If eyes are a little bit obscured, but the segment is still codable, use ‘head turn’. Only code if the duration of the event is >1 sec. |
|  |  | visual-visual | *Both* children look at the monster or parts of the monster, there is *no physical contact* to the monster from any of the children. If eyes are a little bit obscured, but the segment is still codable, use ‘head turn’. Only code if the duration of the event is >1 sec. |

**Additional Analyses**

The pre-registered analyses did not account for the nested structure of the dataset. As participants completed the build task in pairs, their data cannot be considered to be fully independent. Thus, an additional GLMM analysis was run to take this potential confound into account.

A full model of children’s difference scores included predictors of condition (collaborative, individual), society (UK, India) and object (built, identical), the two- and three-way interactions of these variables, gender, and child age in months. Random intercepts for child ID and dyad ID were included to account for the nested structure of the data. A random slope was added to allow for variation in the valuation of each object by dyad. This full model was compared to a null model which included only control predictors (gender, age, random intercepts, and random slope) with a likelihood ratio test. To assess for the contribution of the interaction terms, two reduced models were constructed, one without the three-way interaction, and one without the two-way interactions. These reduced models were compared to the full model using likelihood ratio tests.

**Results**

The full model outperformed the null model (*X*^2^ = 31.44, *df* = 7, *p* < .001), but was not significantly better than the reduced model without the three-way interaction (*X*^2^ = .59, *df* = 1, *p* = .442), or the reduced model without the two-way interactions (*X*^2^ = 4.14, *df* = 3, *p* = .247). Therefore, a model with only main effects was the best fit to the data. Subsequent likelihood ratio tests determined that object was a significant predictor of valuation (*LRC* = 25.78, *p < .*001) but condition (*LRC* = .42, *p* = .519) and society (*LRC* = .51, *p* = .473) were not. There was a trending, but non-significant effect of gender on valuations (*LRC* = 3.19, *p* = .07).

Table S2: Model comparisons. Bold indicates the model with the best fit to the data.

| **Model** | **AIC** | **BIC** | ***p*** |
| --- | --- | --- | --- |
| Null (Age, Gender, Child ID, Dyad ID, Object\|Dyad) | 1235.37 | 1260.18 |  |
| Full (Null + Condition, Society, Object, Condition*Society, Condition*Object, Society*Object, Condition*Society*Object) | 1217.92 | 1267.54 | <.001 vs Null |
| Reduced1 (Full minus Condition*Society*Object) | 1216.51 | 1262.60 | .442 vs Full |
| **Reduced2 (Null + Condition, Society, Object)** | **1214.65** | **1250.11** | **.247 vs Reduced1** |

Table S3: Model summary for the most successful model (Reduced 2).

|  | **Liklihood Ratio** | ***p*** |
| --- | --- | --- |
| Condition (collaborate, individual) | .42 | .519 |
| Society (UK, India) | .51 | .473 |
| Object (built, identical) | 25.78 | <.001 |
| Age | 3.19 | .074 |
| Gender | .66 | .416 |


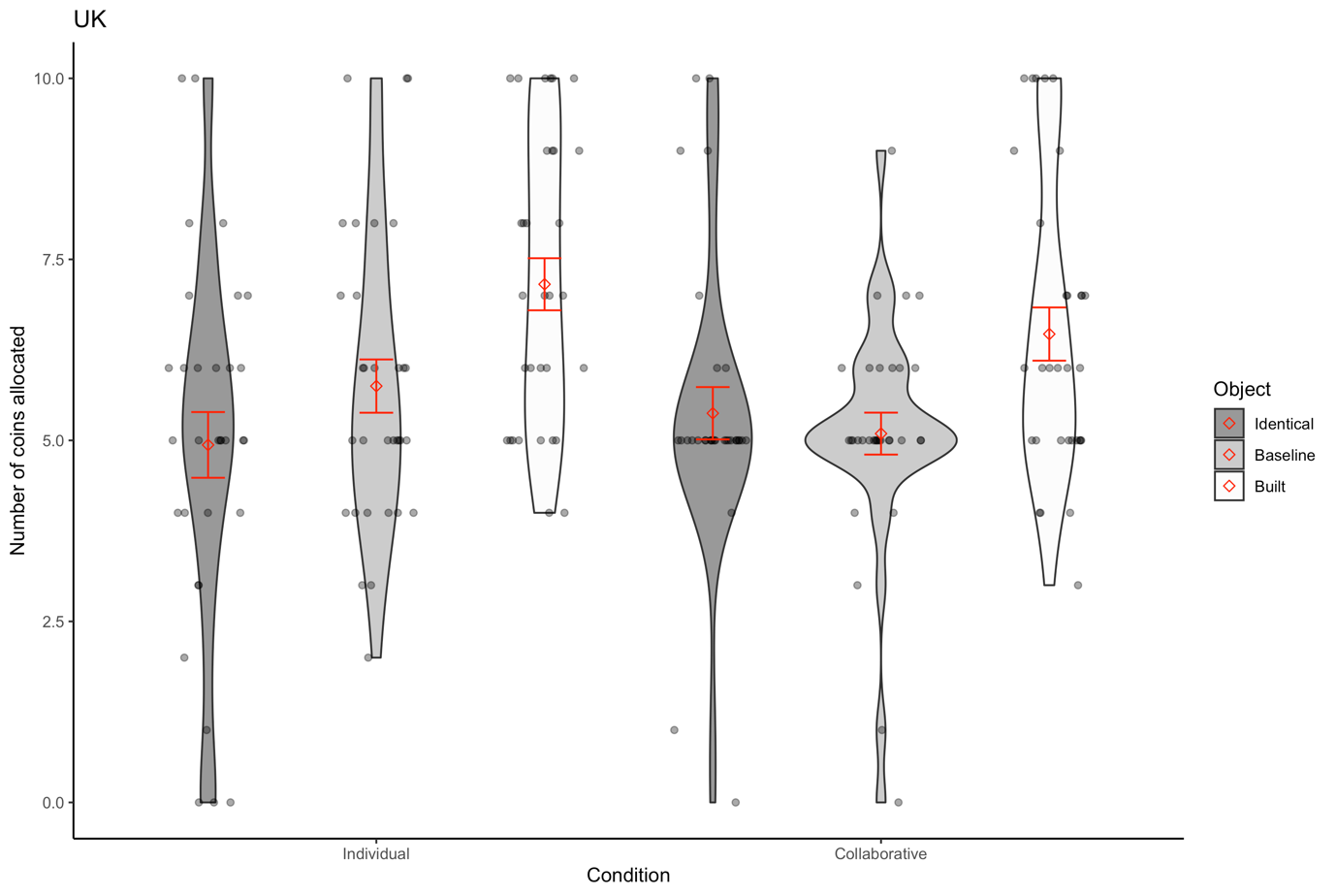


Figure S1: Raw coin allocation data from UK children at baseline (mid-grey), for the identical object (dark-grey) and the built object (light-grey) as a function of collaboration condition Errorbars represent +/-1 S.E.M.


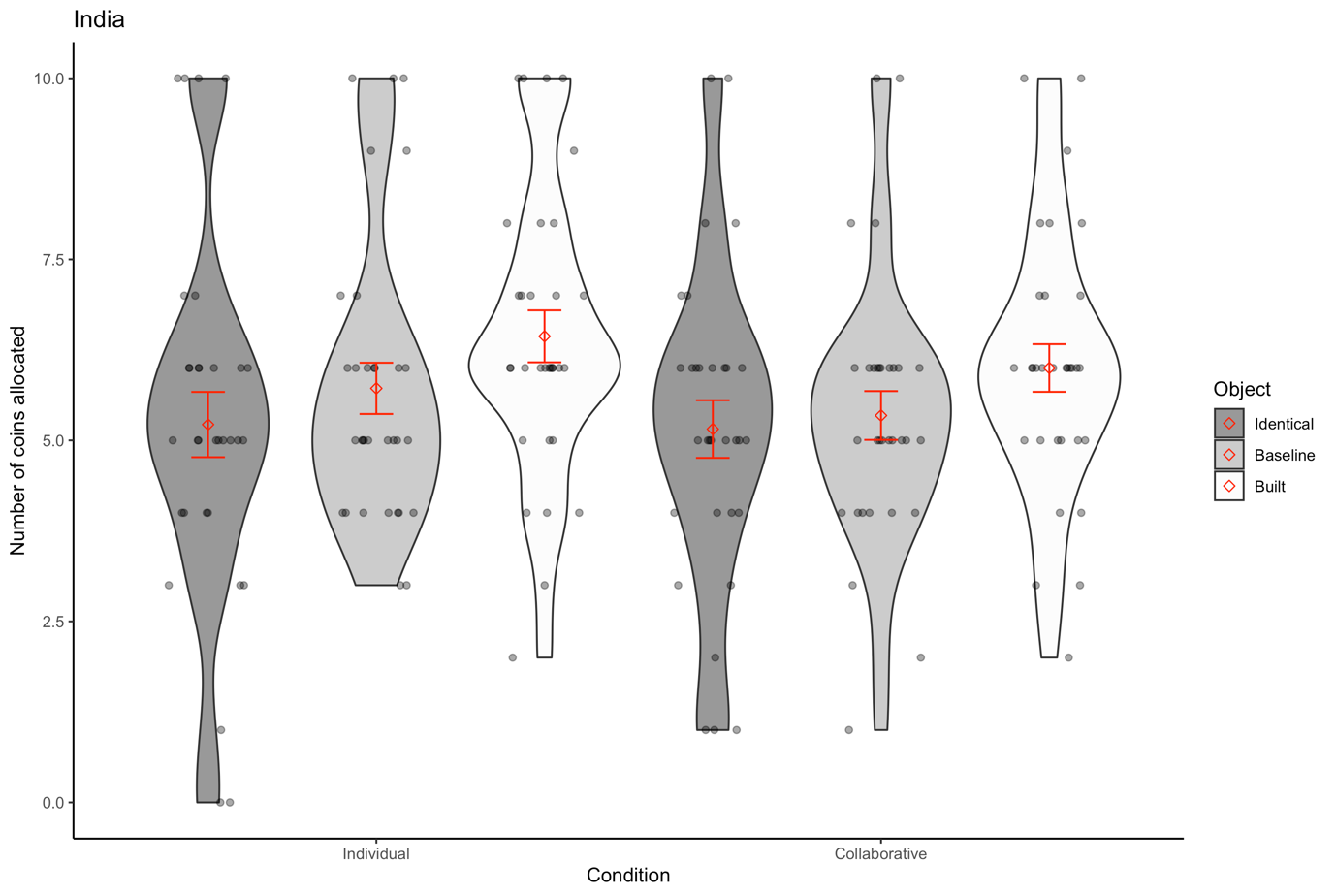


Figure S2: Raw coin allocation data from Indian children at baseline (mid-grey), for the identical object (dark-grey) and the built object (light-grey) as a function of collaboration condition Errorbars represent +/-1 S.E.M.
